# 3-Benzyl-7-chloro-9-phenyl-2-tosyl-2,3,3a,4,9,9a-hexa­hydro-1*H*-pyrrolo[3,4-*b*]quinoline

**DOI:** 10.1107/S1600536809045267

**Published:** 2009-11-04

**Authors:** K. Chinnakali, D. Sudha, M. Jayagobi, R. Raghunathan, Hoong-Kun Fun

**Affiliations:** a Department of Physics, Anna University Chennai, Chennai 600 025, India; b Department of Organic Chemistry, University of Madras, Guindy Campus, Chennai 600 025, India; c X-ray Crystallography Unit, School of Physics, Universiti Sains Malaysia, 11800 USM, Penang, Malaysia

## Abstract

In the title compound, C_31_H_29_ClN_2_O_2_S, the pyrrolidine ring adopts an envelope conformation with the methine C atom adjacent to the NH group as the flap atom. The tetra­hydro­pyridine ring has a half-chair conformation. The two rings are *trans*-fused. The chloro­benzene ring and the adjacent phenyl ring form a dihedral angle of 77.9 (1)°. The benzyl phenyl and the tosyl phenyl rings are oriented at a dihedral angle of 88.0 (1)°. In the crystal, mol­ecules are linked into chains along the *a* axis by N—H⋯Cl and C—H⋯Cl hydrogen bonds and the adjacent chains are cross-linked *via* C—H⋯π inter­actions.

## Related literature

For the caspase-3 inhibitory, vasorelaxing and anti­leukemic activities of pyrroloquinoline compounds, see: Kravchenko *et al.* (2005 ▶); Ferlin *et al.* (2002 ▶); Anderson *et al.* (1988 ▶). For related structures, see: Sudha *et al.* (2007 ▶, 2008*a* ▶,*b* ▶). For the crystal structure of the unchlorinated analogue, see: Chinnakali *et al.* (2009 ▶). For ring puckering parameters, see: Cremer & Pople (1975 ▶). For asymmetry parameters, see: Duax *et al.* (1976 ▶).
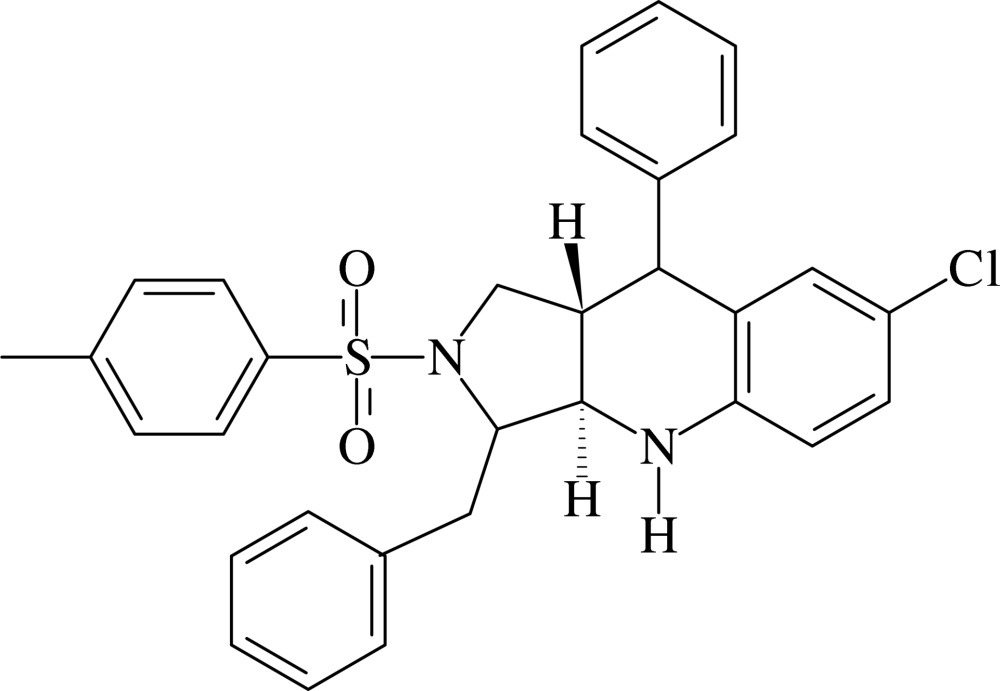


## Experimental

### 

#### Crystal data

C_31_H_29_ClN_2_O_2_S
*M* *_r_* = 529.07
Orthorhombic, 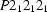

*a* = 10.0106 (1) Å
*b* = 11.8612 (1) Å
*c* = 21.8256 (2) Å
*V* = 2591.52 (4) Å^3^
*Z* = 4
Mo *K*α radiation
μ = 0.26 mm^−1^
*T* = 100 K
0.28 × 0.25 × 0.15 mm

#### Data collection

Bruker SMART APEXII CCD area-detector diffractometer
Absorption correction: multi-scan (*SADABS*; Bruker, 2005 ▶) *T*_min_ = 0.930, *T*_max_ = 0.962
35839 measured reflections
8047 independent reflections
7259 reflections with *I* > 2σ(*I*)
*R*_int_ = 0.045

#### Refinement

*R*[*F* ^2^ > 2σ(*F* ^2^)] = 0.037
*wR*(*F* ^2^) = 0.090
*S* = 1.02
8047 reflections
339 parameters
H atoms treated by a mixture of independent and constrained refinement
Δρ_max_ = 0.42 e Å^−3^
Δρ_min_ = −0.31 e Å^−3^
Absolute structure: Flack (1983 ▶), 3555 Friedel pairs
Flack parameter: 0.02 (4)

### 

Data collection: *APEX2* (Bruker, 2005 ▶); cell refinement: *SAINT* (Bruker, 2005 ▶); data reduction: *SAINT*; program(s) used to solve structure: *SHELXTL* (Sheldrick, 2008 ▶); program(s) used to refine structure: *SHELXTL*; molecular graphics: *SHELXTL*; software used to prepare material for publication: *SHELXTL* and *PLATON* (Spek, 2009 ▶).

## Supplementary Material

Crystal structure: contains datablocks global, I. DOI: 10.1107/S1600536809045267/hb5197sup1.cif

Structure factors: contains datablocks I. DOI: 10.1107/S1600536809045267/hb5197Isup2.hkl

Additional supplementary materials:  crystallographic information; 3D view; checkCIF report

## Figures and Tables

**Table 1:** Hydrogen-bond geometry (Å, °)

*D*—H⋯*A*	*D*—H	H⋯*A*	*D*⋯*A*	*D*—H⋯*A*
N2—H1*N*2⋯Cl1^i^	0.84 (2)	2.83 (2)	3.5947 (13)	153 (2)
C27—H27⋯Cl1^i^	0.93	2.77	3.5980 (16)	149
C17—H17⋯*Cg*2^ii^	0.93	2.70	3.5403 (16)	151
C29—H29⋯*Cg*1^iii^	0.93	2.68	3.6014 (19)	170

Symmetry codes: (i) 
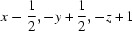
; (ii) 
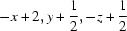
; (iii) 
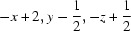
. *Cg*1 and *Cg*2 are the centroids of the C19–C24 and C26–C31 rings, respectively.

**Fig. 1.**
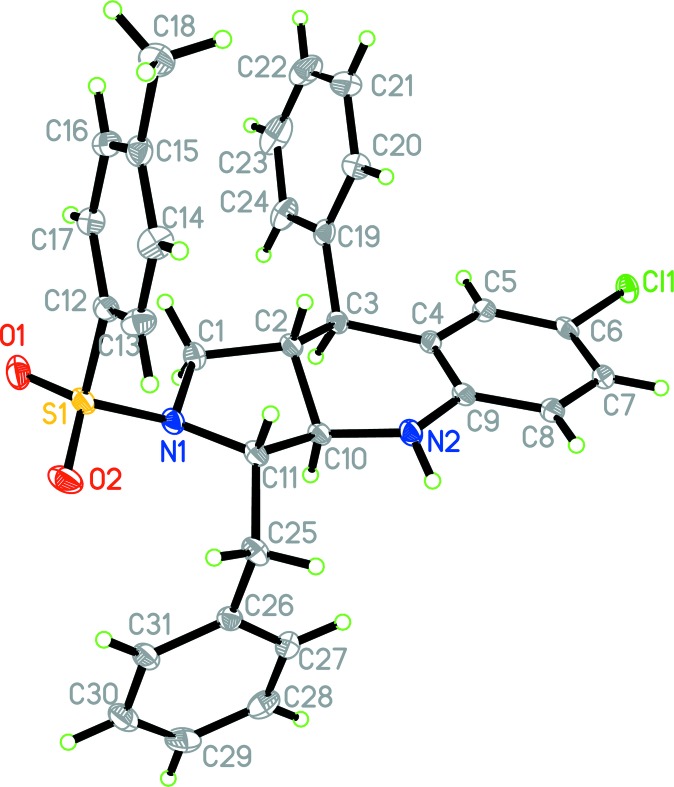
The molecular structure of (I), showing 50% probability displacement ellipsoids. The dashed line indicates a hydrogen bond.

**Fig. 2.**
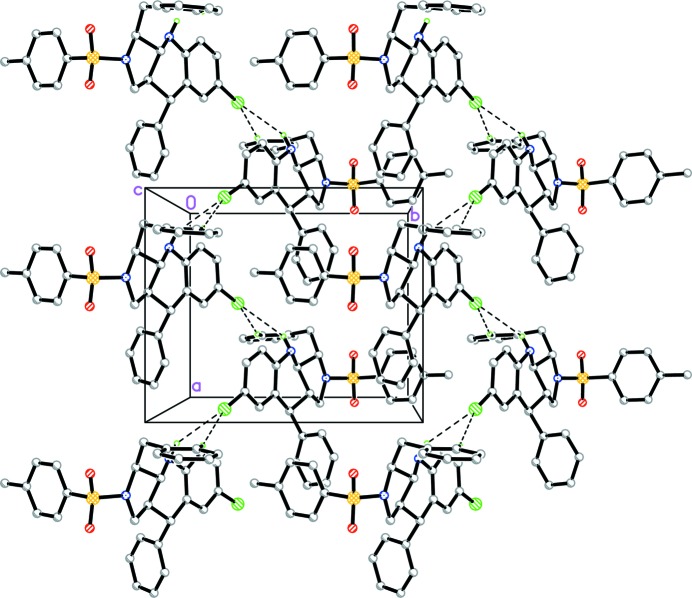
The crystal structure of (I), viewed along the c axis. Hydrogen bonds are shown as dashed lines. For the sake of clarity, H atoms not involved in the interactions have been omitted.

**Fig. 3.**
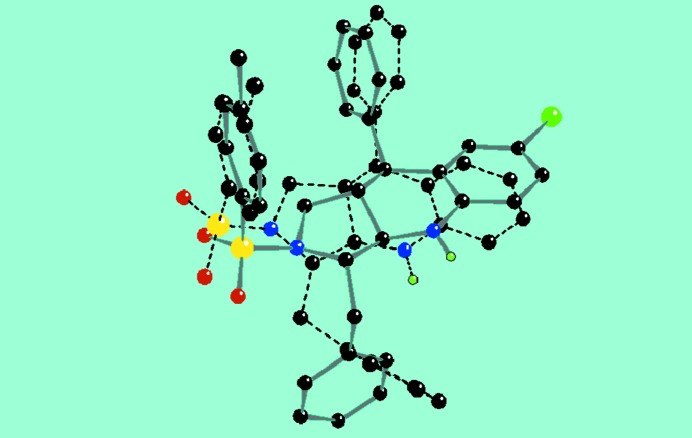
Fit of the title molecule (solid lines) with its unchlorinated analogue (dashed lines). C-bound H atoms have been omitted for clarity.

## supplementary crystallographic information

### Comment

Pyrroloquinoline derivatives have been found to exhibit caspase-3 inhibitory (Kravchenko *et al.*, 2005), vasorelaxing (Ferlin *et al.*, 2002) and antileukemic (Anderson *et al.*, 1988) activities. Previously, we have reported crystal structures of some pyrroloquinoline derivatives (Sudha *et al.*, 2007,2008*a*,b). Now, herein the crystal structure of the title compound is reported.

The pyrrolidine ring adopts an envelope conformation with C10, the envelope flap, lying 0.695 (2) Å out of the plane formed by the rest of the atoms of the ring (r.m.s. deviation 0.019 Å). The asymmetry parameter (Duax *et al.*, 1976) ΔC_s_[C10] = 5.3 (1)° and the puckering parameters (Cremer & Pople, 1975) q_2_ = 0.464 (2) Å and φ = 102.7 (2)°. The tosyl group is attached to the pyrrolidine ring in a equtorial position. The tetrahydropyridine ring adopts a half-chair conformation, with the local twofold rotation axis running through the midpoint of bonds C2—C10 and C4—C9 [asymmetry parameter ΔC_2_[C2—C10] = 3.6 (2)°]. The phenyl ring attached to the tetrahydropyridine ring is in a biaxial position. The dihedral angle between the C4—C9 and C19—C24 rings is 77.9 (1)° and that between the C12—C17 and C26—C31 rings is 88.0 (1)°. Bond lengths and angles are comparable with those in the unchlorinated derivative, 3-benzyl-9-phenyl-2-tosyl-2,3,3a,4,9,9a-hexahydro-1*H*-pyrrolo[3,4-*b*]quinoline (Chinnakali *et al.*, 2009).

The N2—H1N2···Cl1 and C27—H27···Cl1 hydrogen bonds (Table 1) form a pair of bifurcated acceptor bonds, generating a ring of graph-set motif *R*^1^_2_(9). These hydrogen bonds link the molecules into a chain along the *a* axis (Fig. 2). The adjacent chains are crosslinked *via* C—H···π interactions involving the benzyl phenyl and the phenyl ring bound to the fused ring system.

A superposition of the non-H atoms (except the N-bound H atom) of the unchlorinated derivative (Chinnakali *et al.*, 2009) with those of the title molecule using *XP* in *SHELXTL* (Sheldrick, 2008), gave an r.m.s. deviation of 1.27 Å (Fig. 3). In the unchlorinated derivative, the benzyl phenyl ring is oriented in such a way to form an N—H···π interaction. But in the title molecule, the benzyl group is twisted away from the N—H group to make it available for participation in an N—H···Cl hydrogen bond.

### Experimental

InCl_3_ (20 mol%) was added to a mixture of *S*-2-(*N*-cinnamyl-*N*-tosylamino)-3-phenyl propanal (1 mmol) and *p*-chloroaniline (1 mmol) in acetonitrile (20 ml). The reaction mixture was stirred at room temperature for 1 min. On completion of the reaction, as indicated by TLC, the mixture was quenched with water and extracted with ethyl acetate. The organic layer was washed with brine and dried over Na_2_SO_4_. The solvent was evaporated *in vacuo* and the crude product was chromatographed on silica gel using a hexane-ethyl acetate (8.5:1.5 *v*/*v*) mixture to obtain the title compound. The compound was recrystallized from ethyl acetate solution by slow evaporation.

### Refinement

The N-bound H atom was located in a difference map and refined freely [N—H = 0.84 (2) Å]. The remaining H atoms were positioned geometrically (C—H = 0.93–0.98 Å) and allowed to ride on their parent atoms, with *U*_iso_(H) = 1.2*U*_eq_(C) or 1.5*U*_eq_(C_methyl_). A rotating group model was used for methyl groups. Reflection 002 was partially obscured by the beam stop and was omitted.

### Crystal data

**Table:**

C_31_H_29_ClN_2_O_2_S	*F*(000) = 1112
*M**_r_* = 529.07	*D*_x_ = 1.356 Mg m^−^^3^
Orthorhombic, *P*2_1_2_1_2_1_	Mo *K*α radiation, λ = 0.71073 Å
Hall symbol: P 2ac 2ab	Cell parameters from 9900 reflections
*a* = 10.0106 (1) Å	θ = 2.5–29.9°
*b* = 11.8612 (1) Å	µ = 0.26 mm^−^^1^
*c* = 21.8256 (2) Å	*T* = 100 K
*V* = 2591.52 (4) Å^3^	Block, colourless
*Z* = 4	0.28 × 0.25 × 0.15 mm

### Data collection

**Table:**

Bruker SMART APEXII CCD area-detector diffractometer	8047 independent reflections
Radiation source: fine-focus sealed tube	7259 reflections with *I* > 2σ(*I*)
graphite	*R*_int_ = 0.045
φ and ω scans	θ_max_ = 30.7°, θ_min_ = 2.2°
Absorption correction: multi-scan (*SADABS*; Bruker, 2005)	*h* = −13→14
*T*_min_ = 0.930, *T*_max_ = 0.962	*k* = −17→17
35839 measured reflections	*l* = −31→30

### Refinement

**Table:**

Refinement on *F*^2^	Secondary atom site location: difference Fourier map
Least-squares matrix: full	Hydrogen site location: inferred from neighbouring sites
*R*[*F*^2^ > 2σ(*F*^2^)] = 0.037	H atoms treated by a mixture of independent and constrained refinement
*wR*(*F*^2^) = 0.090	*w* = 1/[σ^2^(*F*_o_^2^) + (0.0502*P*)^2^ + 0.3209*P*] where *P* = (*F*_o_^2^ + 2*F*_c_^2^)/3
*S* = 1.02	(Δ/σ)_max_ = 0.001
8047 reflections	Δρ_max_ = 0.42 e Å^−^^3^
339 parameters	Δρ_min_ = −0.31 e Å^−^^3^
0 restraints	Absolute structure: Flack (1983), 3555 Friedel pairs
Primary atom site location: structure-invariant direct methods	Flack parameter: 0.02 (4)

### Special details

**Table:**

Geometry. All e.s.d.'s (except the e.s.d. in the dihedral angle between two l.s. planes) are estimated using the full covariance matrix. The cell e.s.d.'s are taken into account individually in the estimation of e.s.d.'s in distances, angles and torsion angles; correlations between e.s.d.'s in cell parameters are only used when they are defined by crystal symmetry. An approximate (isotropic) treatment of cell e.s.d.'s is used for estimating e.s.d.'s involving l.s. planes.
Refinement. Refinement of *F*^2^ against ALL reflections. The weighted *R*-factor *wR* and goodness of fit *S* are based on *F*^2^, conventional *R*-factors *R* are based on *F*, with *F* set to zero for negative *F*^2^. The threshold expression of *F*^2^ > σ(*F*^2^) is used only for calculating *R*-factors(gt) *etc*. and is not relevant to the choice of reflections for refinement. *R*-factors based on *F*^2^ are statistically about twice as large as those based on *F*, and *R*- factors based on ALL data will be even larger.

### Fractional atomic coordinates and isotropic or equivalent isotropic displacement parameters (Å^2^)

**Table:**

	*x*	*y*	*z*	*U*_iso_*/*U*_eq_	
Cl1	0.99020 (3)	0.23630 (3)	0.611010 (16)	0.01750 (8)	
S1	0.87572 (4)	0.74890 (3)	0.237629 (16)	0.01701 (8)	
O1	1.00459 (12)	0.75400 (9)	0.20897 (5)	0.0229 (2)	
O2	0.75758 (13)	0.75019 (10)	0.20046 (5)	0.0256 (2)	
N1	0.87251 (14)	0.63195 (10)	0.27756 (6)	0.0150 (2)	
N2	0.72707 (13)	0.48831 (11)	0.40955 (6)	0.0159 (2)	
H1N2	0.654 (2)	0.4565 (17)	0.4026 (10)	0.027 (5)*	
C1	0.99589 (16)	0.60584 (12)	0.31441 (7)	0.0160 (3)	
H1A	1.0509	0.6725	0.3196	0.019*	
H1B	1.0486	0.5472	0.2950	0.019*	
C2	0.94008 (14)	0.56589 (12)	0.37568 (7)	0.0137 (3)	
H2	0.9205	0.6320	0.4011	0.016*	
C3	1.02378 (14)	0.48294 (12)	0.41319 (7)	0.0132 (3)	
H3	1.0572	0.4246	0.3853	0.016*	
C4	0.93338 (14)	0.42593 (12)	0.46062 (7)	0.0131 (3)	
C5	0.99179 (15)	0.36541 (11)	0.50869 (7)	0.0142 (3)	
H5	1.0843	0.3619	0.5118	0.017*	
C6	0.91344 (15)	0.31068 (12)	0.55164 (7)	0.0141 (3)	
C7	0.77481 (15)	0.31665 (12)	0.54938 (7)	0.0149 (3)	
H7	0.7228	0.2811	0.5789	0.018*	
C8	0.71615 (15)	0.37664 (13)	0.50226 (7)	0.0155 (3)	
H8	0.6235	0.3817	0.5004	0.019*	
C9	0.79311 (14)	0.43009 (12)	0.45709 (7)	0.0134 (3)	
C10	0.80912 (14)	0.51222 (12)	0.35619 (7)	0.0133 (3)	
H10	0.8266	0.4425	0.3335	0.016*	
C11	0.75027 (16)	0.60125 (12)	0.31332 (7)	0.0147 (3)	
H11	0.7237	0.6666	0.3379	0.018*	
C12	0.86417 (16)	0.86286 (11)	0.28916 (7)	0.0159 (3)	
C13	0.73961 (16)	0.90828 (13)	0.30416 (8)	0.0201 (3)	
H13	0.6617	0.8776	0.2880	0.024*	
C14	0.73412 (16)	0.99983 (13)	0.34358 (8)	0.0212 (3)	
H14	0.6516	1.0306	0.3537	0.025*	
C15	0.85024 (15)	1.04677 (12)	0.36832 (7)	0.0170 (3)	
C16	0.97308 (15)	0.99944 (12)	0.35296 (7)	0.0175 (3)	
H16	1.0509	1.0297	0.3694	0.021*	
C17	0.98130 (15)	0.90773 (12)	0.31342 (7)	0.0157 (3)	
H17	1.0638	0.8768	0.3033	0.019*	
C18	0.84443 (18)	1.14674 (14)	0.41044 (8)	0.0236 (3)	
H18A	0.9244	1.1907	0.4059	0.035*	
H18B	0.8369	1.1212	0.4520	0.035*	
H18C	0.7683	1.1922	0.4003	0.035*	
C19	1.14346 (15)	0.53881 (12)	0.44351 (7)	0.0158 (3)	
C20	1.12450 (17)	0.61970 (14)	0.48937 (7)	0.0217 (3)	
H20	1.0382	0.6402	0.5006	0.026*	
C21	1.2328 (2)	0.66992 (16)	0.51843 (9)	0.0292 (4)	
H21	1.2188	0.7245	0.5483	0.035*	
C22	1.36117 (19)	0.63871 (17)	0.50286 (9)	0.0324 (4)	
H22	1.4338	0.6714	0.5227	0.039*	
C23	1.38142 (17)	0.55904 (16)	0.45789 (10)	0.0318 (4)	
H23	1.4680	0.5380	0.4474	0.038*	
C24	1.27344 (16)	0.50968 (14)	0.42795 (8)	0.0221 (3)	
H24	1.2885	0.4568	0.3973	0.027*	
C25	0.62882 (16)	0.56231 (13)	0.27564 (7)	0.0171 (3)	
H25A	0.5588	0.5405	0.3040	0.020*	
H25B	0.5961	0.6264	0.2525	0.020*	
C26	0.65082 (14)	0.46589 (12)	0.23147 (7)	0.0150 (3)	
C27	0.65518 (16)	0.35343 (13)	0.25089 (7)	0.0192 (3)	
H27	0.6466	0.3369	0.2924	0.023*	
C28	0.67212 (16)	0.26635 (13)	0.20928 (8)	0.0237 (3)	
H28	0.6760	0.1922	0.2230	0.028*	
C29	0.68336 (18)	0.28986 (14)	0.14704 (8)	0.0253 (4)	
H29	0.6945	0.2315	0.1190	0.030*	
C30	0.67796 (17)	0.40097 (14)	0.12689 (8)	0.0239 (3)	
H30	0.6850	0.4171	0.0853	0.029*	
C31	0.66208 (16)	0.48796 (13)	0.16892 (7)	0.0189 (3)	
H31	0.6589	0.5621	0.1551	0.023*	

### Atomic displacement parameters (Å^2^)

**Table:**

	*U*^11^	*U*^22^	*U*^33^	*U*^12^	*U*^13^	*U*^23^
Cl1	0.01853 (15)	0.02012 (16)	0.01386 (15)	0.00295 (13)	0.00000 (12)	0.00457 (12)
S1	0.02840 (18)	0.01221 (14)	0.01041 (15)	−0.00087 (15)	−0.00294 (13)	0.00055 (13)
O1	0.0372 (6)	0.0165 (5)	0.0151 (5)	−0.0010 (6)	0.0072 (5)	0.0006 (4)
O2	0.0404 (6)	0.0180 (5)	0.0185 (5)	−0.0022 (5)	−0.0136 (5)	0.0016 (5)
N1	0.0210 (6)	0.0123 (5)	0.0117 (6)	0.0001 (5)	−0.0011 (5)	0.0020 (4)
N2	0.0138 (6)	0.0219 (6)	0.0120 (6)	−0.0002 (5)	−0.0011 (5)	0.0026 (5)
C1	0.0179 (7)	0.0172 (6)	0.0130 (7)	0.0009 (6)	0.0002 (6)	0.0031 (5)
C2	0.0168 (6)	0.0134 (6)	0.0108 (7)	0.0004 (5)	−0.0010 (5)	0.0000 (5)
C3	0.0155 (6)	0.0127 (6)	0.0114 (6)	0.0007 (5)	0.0016 (5)	−0.0006 (5)
C4	0.0146 (6)	0.0124 (6)	0.0121 (7)	−0.0003 (5)	0.0012 (5)	−0.0013 (5)
C5	0.0144 (6)	0.0142 (6)	0.0139 (7)	0.0004 (5)	−0.0002 (5)	−0.0003 (5)
C6	0.0188 (7)	0.0140 (6)	0.0096 (7)	0.0020 (5)	−0.0031 (5)	−0.0001 (5)
C7	0.0164 (7)	0.0161 (6)	0.0121 (7)	−0.0012 (5)	0.0015 (5)	−0.0011 (5)
C8	0.0148 (6)	0.0174 (7)	0.0145 (7)	−0.0009 (5)	0.0002 (5)	−0.0015 (5)
C9	0.0155 (6)	0.0138 (6)	0.0109 (7)	0.0006 (5)	0.0003 (5)	−0.0021 (5)
C10	0.0162 (6)	0.0129 (6)	0.0109 (7)	0.0004 (5)	−0.0014 (5)	0.0002 (5)
C11	0.0187 (7)	0.0141 (6)	0.0112 (7)	0.0016 (5)	−0.0005 (6)	−0.0008 (5)
C12	0.0227 (7)	0.0122 (6)	0.0129 (7)	−0.0002 (5)	−0.0008 (6)	0.0000 (5)
C13	0.0178 (7)	0.0176 (7)	0.0250 (9)	−0.0002 (6)	−0.0048 (6)	−0.0004 (6)
C14	0.0179 (7)	0.0186 (7)	0.0272 (8)	0.0028 (6)	0.0003 (6)	−0.0009 (6)
C15	0.0203 (7)	0.0145 (6)	0.0163 (7)	0.0007 (5)	0.0030 (6)	0.0005 (5)
C16	0.0177 (7)	0.0174 (7)	0.0175 (7)	−0.0021 (5)	−0.0002 (5)	−0.0018 (6)
C17	0.0156 (7)	0.0145 (6)	0.0171 (7)	0.0020 (5)	−0.0002 (6)	−0.0001 (5)
C18	0.0277 (8)	0.0205 (7)	0.0225 (8)	0.0014 (6)	0.0042 (7)	−0.0066 (6)
C19	0.0164 (7)	0.0158 (6)	0.0152 (7)	−0.0012 (5)	−0.0012 (5)	0.0054 (5)
C20	0.0224 (8)	0.0236 (7)	0.0190 (8)	−0.0030 (7)	−0.0015 (6)	−0.0008 (6)
C21	0.0353 (10)	0.0311 (9)	0.0211 (9)	−0.0126 (8)	−0.0070 (7)	−0.0008 (7)
C22	0.0266 (9)	0.0339 (9)	0.0366 (11)	−0.0130 (8)	−0.0166 (8)	0.0158 (8)
C23	0.0153 (7)	0.0314 (9)	0.0486 (12)	0.0014 (7)	−0.0031 (8)	0.0182 (8)
C24	0.0174 (7)	0.0178 (7)	0.0311 (9)	0.0020 (6)	0.0026 (6)	0.0071 (6)
C25	0.0182 (7)	0.0201 (6)	0.0129 (7)	0.0040 (6)	−0.0020 (6)	0.0005 (5)
C26	0.0135 (6)	0.0176 (6)	0.0139 (7)	−0.0005 (5)	−0.0010 (5)	−0.0008 (5)
C27	0.0188 (7)	0.0206 (7)	0.0181 (8)	−0.0038 (6)	0.0006 (6)	0.0030 (6)
C28	0.0229 (7)	0.0157 (7)	0.0325 (9)	−0.0040 (6)	−0.0003 (7)	0.0000 (6)
C29	0.0283 (8)	0.0223 (7)	0.0253 (9)	−0.0029 (6)	−0.0037 (7)	−0.0103 (6)
C30	0.0297 (8)	0.0273 (8)	0.0148 (8)	−0.0015 (7)	−0.0033 (6)	−0.0037 (6)
C31	0.0234 (7)	0.0194 (7)	0.0141 (7)	−0.0009 (6)	−0.0039 (6)	0.0011 (6)

### Geometric parameters (Å, °)

**Table:**

Cl1—C6	1.7459 (15)	C14—C15	1.397 (2)
S1—O2	1.4341 (12)	C14—H14	0.93
S1—O1	1.4349 (12)	C15—C16	1.393 (2)
S1—N1	1.6386 (12)	C15—C18	1.502 (2)
S1—C12	1.7623 (15)	C16—C17	1.391 (2)
N1—C11	1.496 (2)	C16—H16	0.93
N1—C1	1.506 (2)	C17—H17	0.93
N2—C9	1.4107 (19)	C18—H18A	0.96
N2—C10	1.4531 (19)	C18—H18B	0.96
N2—H1N2	0.84 (2)	C18—H18C	0.96
C1—C2	1.525 (2)	C19—C24	1.388 (2)
C1—H1A	0.97	C19—C20	1.399 (2)
C1—H1B	0.97	C20—C21	1.390 (2)
C2—C10	1.518 (2)	C20—H20	0.93
C2—C3	1.530 (2)	C21—C22	1.380 (3)
C2—H2	0.98	C21—H21	0.93
C3—C19	1.521 (2)	C22—C23	1.377 (3)
C3—C4	1.532 (2)	C22—H22	0.93
C3—H3	0.98	C23—C24	1.392 (3)
C4—C5	1.399 (2)	C23—H23	0.93
C4—C9	1.4072 (19)	C24—H24	0.93
C5—C6	1.384 (2)	C25—C26	1.512 (2)
C5—H5	0.93	C25—H25A	0.97
C6—C7	1.390 (2)	C25—H25B	0.97
C7—C8	1.382 (2)	C26—C31	1.394 (2)
C7—H7	0.93	C26—C27	1.400 (2)
C8—C9	1.403 (2)	C27—C28	1.386 (2)
C8—H8	0.93	C27—H27	0.93
C10—C11	1.529 (2)	C28—C29	1.391 (3)
C10—H10	0.98	C28—H28	0.93
C11—C25	1.539 (2)	C29—C30	1.390 (2)
C11—H11	0.98	C29—H29	0.93
C12—C17	1.392 (2)	C30—C31	1.390 (2)
C12—C13	1.397 (2)	C30—H30	0.93
C13—C14	1.386 (2)	C31—H31	0.93
C13—H13	0.93		
			
O2—S1—O1	119.63 (7)	C14—C13—C12	118.86 (15)
O2—S1—N1	107.08 (7)	C14—C13—H13	120.6
O1—S1—N1	106.57 (7)	C12—C13—H13	120.6
O2—S1—C12	107.38 (7)	C13—C14—C15	121.25 (15)
O1—S1—C12	107.75 (7)	C13—C14—H14	119.4
N1—S1—C12	107.97 (7)	C15—C14—H14	119.4
C11—N1—C1	110.00 (11)	C16—C15—C14	118.75 (14)
C11—N1—S1	119.97 (10)	C16—C15—C18	119.99 (14)
C1—N1—S1	116.23 (10)	C14—C15—C18	121.27 (14)
C9—N2—C10	114.84 (12)	C17—C16—C15	121.11 (14)
C9—N2—H1N2	108.8 (14)	C17—C16—H16	119.4
C10—N2—H1N2	115.8 (14)	C15—C16—H16	119.4
N1—C1—C2	103.40 (12)	C16—C17—C12	119.01 (14)
N1—C1—H1A	111.1	C16—C17—H17	120.5
C2—C1—H1A	111.1	C12—C17—H17	120.5
N1—C1—H1B	111.1	C15—C18—H18A	109.5
C2—C1—H1B	111.1	C15—C18—H18B	109.5
H1A—C1—H1B	109.0	H18A—C18—H18B	109.5
C10—C2—C1	101.60 (12)	C15—C18—H18C	109.5
C10—C2—C3	110.69 (11)	H18A—C18—H18C	109.5
C1—C2—C3	117.94 (12)	H18B—C18—H18C	109.5
C10—C2—H2	108.7	C24—C19—C20	118.21 (15)
C1—C2—H2	108.7	C24—C19—C3	121.56 (14)
C3—C2—H2	108.7	C20—C19—C3	120.21 (13)
C19—C3—C2	112.59 (11)	C21—C20—C19	120.94 (17)
C19—C3—C4	111.32 (12)	C21—C20—H20	119.5
C2—C3—C4	108.78 (12)	C19—C20—H20	119.5
C19—C3—H3	108.0	C22—C21—C20	119.93 (18)
C2—C3—H3	108.0	C22—C21—H21	120.0
C4—C3—H3	108.0	C20—C21—H21	120.0
C5—C4—C9	118.43 (13)	C23—C22—C21	119.78 (17)
C5—C4—C3	119.09 (13)	C23—C22—H22	120.1
C9—C4—C3	122.47 (13)	C21—C22—H22	120.1
C6—C5—C4	120.78 (14)	C22—C23—C24	120.57 (17)
C6—C5—H5	119.6	C22—C23—H23	119.7
C4—C5—H5	119.6	C24—C23—H23	119.7
C5—C6—C7	121.18 (14)	C19—C24—C23	120.54 (16)
C5—C6—Cl1	119.37 (11)	C19—C24—H24	119.7
C7—C6—Cl1	119.42 (12)	C23—C24—H24	119.7
C8—C7—C6	118.47 (14)	C26—C25—C11	116.91 (13)
C8—C7—H7	120.8	C26—C25—H25A	108.1
C6—C7—H7	120.8	C11—C25—H25A	108.1
C7—C8—C9	121.50 (14)	C26—C25—H25B	108.1
C7—C8—H8	119.2	C11—C25—H25B	108.1
C9—C8—H8	119.2	H25A—C25—H25B	107.3
C8—C9—C4	119.59 (14)	C31—C26—C27	118.21 (14)
C8—C9—N2	118.73 (13)	C31—C26—C25	119.60 (13)
C4—C9—N2	121.67 (13)	C27—C26—C25	122.14 (14)
N2—C10—C2	110.21 (12)	C28—C27—C26	121.02 (15)
N2—C10—C11	114.05 (12)	C28—C27—H27	119.5
C2—C10—C11	102.39 (11)	C26—C27—H27	119.5
N2—C10—H10	110.0	C27—C28—C29	120.02 (15)
C2—C10—H10	110.0	C27—C28—H28	120.0
C11—C10—H10	110.0	C29—C28—H28	120.0
N1—C11—C10	99.92 (12)	C30—C29—C28	119.71 (16)
N1—C11—C25	116.13 (12)	C30—C29—H29	120.1
C10—C11—C25	115.10 (12)	C28—C29—H29	120.1
N1—C11—H11	108.4	C31—C30—C29	119.96 (16)
C10—C11—H11	108.4	C31—C30—H30	120.0
C25—C11—H11	108.4	C29—C30—H30	120.0
C17—C12—C13	121.01 (13)	C30—C31—C26	121.07 (14)
C17—C12—S1	118.72 (12)	C30—C31—H31	119.5
C13—C12—S1	120.25 (12)	C26—C31—H31	119.5
			
O2—S1—N1—C11	−50.80 (12)	C2—C10—C11—N1	43.40 (13)
O1—S1—N1—C11	−179.92 (10)	N2—C10—C11—C25	−72.42 (16)
C12—S1—N1—C11	64.55 (12)	C2—C10—C11—C25	168.55 (12)
O2—S1—N1—C1	172.80 (10)	O2—S1—C12—C17	−155.56 (12)
O1—S1—N1—C1	43.67 (12)	O1—S1—C12—C17	−25.46 (14)
C12—S1—N1—C1	−71.85 (12)	N1—S1—C12—C17	89.29 (13)
C11—N1—C1—C2	−3.90 (14)	O2—S1—C12—C13	23.14 (15)
S1—N1—C1—C2	136.63 (10)	O1—S1—C12—C13	153.24 (12)
N1—C1—C2—C10	30.84 (14)	N1—S1—C12—C13	−92.01 (13)
N1—C1—C2—C3	151.97 (12)	C17—C12—C13—C14	0.4 (2)
C10—C2—C3—C19	−169.50 (12)	S1—C12—C13—C14	−178.31 (12)
C1—C2—C3—C19	74.18 (16)	C12—C13—C14—C15	−0.1 (2)
C10—C2—C3—C4	−45.62 (15)	C13—C14—C15—C16	−0.4 (2)
C1—C2—C3—C4	−161.95 (12)	C13—C14—C15—C18	179.29 (15)
C19—C3—C4—C5	−41.60 (17)	C14—C15—C16—C17	0.5 (2)
C2—C3—C4—C5	−166.23 (12)	C18—C15—C16—C17	−179.16 (14)
C19—C3—C4—C9	139.71 (14)	C15—C16—C17—C12	−0.2 (2)
C2—C3—C4—C9	15.08 (19)	C13—C12—C17—C16	−0.2 (2)
C9—C4—C5—C6	0.2 (2)	S1—C12—C17—C16	178.46 (11)
C3—C4—C5—C6	−178.56 (13)	C2—C3—C19—C24	−115.72 (15)
C4—C5—C6—C7	−1.8 (2)	C4—C3—C19—C24	121.83 (15)
C4—C5—C6—Cl1	−179.87 (11)	C2—C3—C19—C20	65.90 (17)
C5—C6—C7—C8	1.5 (2)	C4—C3—C19—C20	−56.56 (17)
Cl1—C6—C7—C8	179.53 (11)	C24—C19—C20—C21	0.2 (2)
C6—C7—C8—C9	0.5 (2)	C3—C19—C20—C21	178.68 (15)
C7—C8—C9—C4	−2.1 (2)	C19—C20—C21—C22	−1.1 (3)
C7—C8—C9—N2	178.85 (14)	C20—C21—C22—C23	1.0 (3)
C5—C4—C9—C8	1.7 (2)	C21—C22—C23—C24	0.0 (3)
C3—C4—C9—C8	−179.61 (12)	C20—C19—C24—C23	0.7 (2)
C5—C4—C9—N2	−179.25 (12)	C3—C19—C24—C23	−177.67 (14)
C3—C4—C9—N2	−0.5 (2)	C22—C23—C24—C19	−0.9 (2)
C10—N2—C9—C8	−162.78 (13)	N1—C11—C25—C26	54.31 (17)
C10—N2—C9—C4	18.1 (2)	C10—C11—C25—C26	−61.90 (18)
C9—N2—C10—C2	−49.72 (16)	C11—C25—C26—C31	−103.37 (17)
C9—N2—C10—C11	−164.22 (12)	C11—C25—C26—C27	79.21 (19)
C1—C2—C10—N2	−168.63 (12)	C31—C26—C27—C28	0.9 (2)
C3—C2—C10—N2	65.30 (15)	C25—C26—C27—C28	178.35 (14)
C1—C2—C10—C11	−46.93 (14)	C26—C27—C28—C29	−0.8 (2)
C3—C2—C10—C11	−172.99 (11)	C27—C28—C29—C30	0.2 (3)
C1—N1—C11—C10	−24.29 (13)	C28—C29—C30—C31	0.3 (3)
S1—N1—C11—C10	−163.12 (9)	C29—C30—C31—C26	−0.3 (3)
C1—N1—C11—C25	−148.72 (12)	C27—C26—C31—C30	−0.4 (2)
S1—N1—C11—C25	72.44 (15)	C25—C26—C31—C30	−177.87 (15)
N2—C10—C11—N1	162.44 (11)		

### Hydrogen-bond geometry (Å, °)

**Table:**

*D*—H···*A*	*D*—H	H···*A*	*D*···*A*	*D*—H···*A*
N2—H1N2···Cl1^i^	0.84 (2)	2.83 (2)	3.5947 (13)	153 (2)
C25—H25B···O2	0.97	2.46	3.0529 (19)	119
C27—H27···Cl1^i^	0.93	2.77	3.5980 (16)	149
C17—H17···Cg2^ii^	0.93	2.70	3.5403 (16)	151
C29—H29···Cg1^iii^	0.93	2.68	3.6014 (19)	170

Symmetry codes: (i) *x*−1/2, −*y*+1/2, −*z*+1; (ii) −*x*+2, *y*+1/2, −*z*+1/2; (iii) −*x*+2, *y*−1/2, −*z*+1/2.

## References

[bb1] Anderson, W. K., Heider, A. R., Raju, N. & Yucht, J. A. (1988). *J. Med. Chem.* **31**, 2097–2102. 10.1021/jm00119a008 3184121

[bb2] Bruker (2005). *APEX2*, *SAINT* and *SADABS*. Bruker AXS Inc., Madison, Wisconsin, USA.

[bb3] Chinnakali, K., Sudha, D., Jayagobi, M., Raghunathan, R. & Fun, H.-K. (2009). *Acta Cryst.* E**65**, o2923. 10.1107/S1600536809044547 PMC2971326 21578501

[bb4] Cremer, D. & Pople, J. A. (1975). *J. Am. Chem. Soc.* **97**, 1354–1358.

[bb5] Duax, W. L., Weeks, C. M. & Rohrer, D. C. (1976). *Topics in Stereochemistry*, Vol. 9, edited by E. L. Eliel & N. L. Allinger, pp. 271–383. New York: John Wiley.

[bb6] Ferlin, M. G., Chiarelotto, G., Antonucci, F., Caparrotta, L. & Froldi, G. (2002). *Eur. J. Med. Chem.* **37**, 427–434. 10.1016/s0223-5234(02)01355-7 12008057

[bb7] Flack, H. D. (1983). *Acta Cryst.* A**39**, 876–881.

[bb8] Kravchenko, D. V., Kysil, V. M., Tkachenko, S. E., Maliarchouk, S., Okun, I. M. & Ivachtchenko, A. V. (2005). *Il Farm* **60**, 804–809. 10.1016/j.farmac.2005.08.001 16182295

[bb9] Sheldrick, G. M. (2008). *Acta Cryst.* A**64**, 112–122. 10.1107/S0108767307043930 18156677

[bb10] Spek, A. L. (2009). *Acta Cryst.* D**65**, 148–155. 10.1107/S090744490804362X PMC2631630 19171970

[bb11] Sudha, D., Chinnakali, K., Jayagobi, M., Raghunathan, R. & Fun, H.-K. (2007). *Acta Cryst.* E**63**, o4914–o4915.

[bb12] Sudha, D., Chinnakali, K., Jayagobi, M., Raghunathan, R. & Fun, H.-K. (2008*a*). *Acta Cryst.* E**64**, o134. 10.1107/S1600536807063222 PMC2915203 21200698

[bb13] Sudha, D., Chinnakali, K., Jayagobi, M., Raghunathan, R. & Fun, H.-K. (2008*b*). *Acta Cryst.* E**64**, o425. 10.1107/S1600536808000482 PMC2960215 21201452

